# Biological characteristics and functions of a novel glutamate dehydrogenase from *Trichinella spiralis*[Fn FN2]

**DOI:** 10.1051/parasite/2024065

**Published:** 2024-10-28

**Authors:** Yong Kang Cheng, Yao Zhang, Zhao Yu Zhang, Pei Kun Cong, Ji Yu Feng, Ru Zhang, Shao Rong Long, Xi Zhang, Zhong Quan Wang, Jing Cui

**Affiliations:** Department of Parasitology, School of Basic Medical Sciences, Zhengzhou University Zhengzhou 450052 China

**Keywords:** *Trichinella spiralis*, Glutamate dehydrogenase, RNA interference, Metabolism, Molting

## Abstract

Glutamate dehydrogenase (GDH) plays an important role in the metabolism of organisms. Its high abundance in mitochondria in particular highlights its core role in cellular physiological processes. GDH catalyzes the mutual conversion between L-glutamic acid and α-ketoglutaric acids. At the same time, this transformation is accompanied by the oxidation-reduction of NAD(H) or NADP(H). This process not only helps to link amino acid metabolism with sugar metabolism, but also helps maintain the balance of intracellular pH and nitrogen homeostasis. In this study, a novel *Trichinella spiralis* glutamate dehydrogenase (TsGDH) was cloned, expressed and identified. The results revealed that TsGDH was expressed at various stages of development of the nematode *T. spiralis*, with higher expression levels in the adult worm stage, and was mainly localized in the cuticle, muscular layer, stichosome and female intrauterine embryos. After RNAi treatment, larval natural TsGDH enzyme activity was obviously reduced, and metabolism, molting, growth and reproduction were also significantly inhibited. The results indicate that TsGDH plays an important role in the development and survival of *T. spiralis*, and it may be a potential molecular target of anti-*Trichinella* vaccines and drugs.

## Abstract (fr)

La glutamate déshydrogénase (GDH) joue un rôle important dans le métabolisme des organismes. En particulier, sa forte abondance dans les mitochondries souligne son rôle essentiel dans les processus physiologiques cellulaires. La GDH catalyse la conversion mutuelle entre l’acide L-glutamique et les acides α-cétoglutariques. Dans le même temps, cette transformation s’accompagne de l’oxydoréduction du NAD(H) ou du NADP(H). Ce processus permet non seulement de lier le métabolisme des acides aminés au métabolisme du sucre, mais également de maintenir l’équilibre du pH intracellulaire et l’homéostasie de l’azote. Dans cette étude, une nouvelle glutamate déshydrogénase de *Trichinella spiralis* (TsGDH) a été clonée, exprimée et identifiée. Les résultats ont révélé que la TsGDH était exprimée à différents stades de développement du nématode *T. spiralis*, avec un niveau d’expression plus élevé au stade adulte du ver, et qu’elle est principalement localisée dans la cuticule, la couche musculaire, le stichosome et les embryons intra-utérins chez les femelles. Après traitement par ARN interférent, l’activité enzymatique naturelle de la TsGDH des larves était réduite, et le métabolisme, la mue, la croissance et la reproduction étaient également significativement inhibés. Les résultats indiquent que la TsGDH joue un rôle important dans le développement et la survie de *T. spiralis*, et qu’elle pourrait être une cible moléculaire potentielle pour un vaccin et des médicaments anti-*Trichinella*.

## Introduction

Trichinellosis is an important zoonotic parasitic disease caused by consuming raw or undercooked meat from pigs and other animals infected with *Trichinella* spp. [9]. *Trichinella spiralis* (Owen, 1835), the most common causative agent of trichinellosis, is distributed worldwide [37]. From 2009 to 2020, eight outbreaks of human trichinellosis were recorded in the southwest region of China and involved 479 cases and 2 deaths [76]. Pork is the main source of trichinellosis outbreaks [24, 44]. *Trichinella* infection not only poses a serious threat to public health, but also represents a significant challenge in the food safety of meat products [3, 54]. Since *T. spiralis* is a multicellular parasitic nematode with a complex life cycle, and each worm stage has stage-specific antigens, immune response produced by vaccination with an individual recombinant *T. spiralis* protein (paramyosin, calreticulin, Nudix hydrolase, glutathione S-transferase, aminopeptidase, Ts31, diverse serine proteases and cathepsin, galectin, etc.) was not enough to entirely prevent larva challenge infection. The currently known anti-*Trichinella* vaccines are unable to completely eliminate infective muscle larvae (ML) from vaccinated animals after challenge [1, 63]. Hence, it is necessary to develop novel anti-*Trichinella* vaccines to control *Trichinella* infection in domestic animals reared as a source of food.

After infective ML encapsulated in meat are ingested and digested by gastric juices, the ML are liberated from their collagen capsules and develop into intestinal infective larvae (IIL) under the stimulation of bile and enteral contents, and the IIL penetrate into the enteral epithelium where they molt 4 times to grow into adult worms (AWs) at 31 hours post infection (hpi) [42]. The male and female adults mate within gut mucosal epithelia and produce newborn larvae (NBL). NBL enter the bloodstream via the gut mucosal blood capillary and arrive in skeletal muscle where they are encapsulated and complete the *T. spiralis* life cycle [7, 43]. During growth and development, the *T. spiralis* worm obtains various forms of energy for its survival through glycometabolism, and lipid and protein metabolism; it also has a complete citric acid cycle [11, 70].

Glutamate dehydrogenase (GDH) is an enzyme widely present in organisms and plays a key role in cellular energy metabolism [19]. GDH participates in important physiological processes such as the tricarboxylic acid cycle, ammonia metabolism regulation, signal transduction and energy production [8]. This enzyme, with nicotinamide adenine dinucleotide (NADH) or nicotinamide adenine dinucleotide phosphate (NADPH) as coenzymes, mainly catalyzes the reversible conversion between L-glutamic acid and α-ketoglutaric acid. GDH can synergistically act with aminotransferase and participate in amino acid metabolism in organisms [33]. Aminotransferase transfers the amino acids of other amino acids to α-ketoglutarate to generate L-glutamic acid, which is then deaminated by glutamate dehydrogenase to produce NH_3_, thereby completing the deamination process in amino acid catabolism. After deamination of amino acids, α-ketoglutaric acid is generated by L-glutamic acid and acts as an intermediate product in the tricarboxylic acid cycle, entering the tricarboxylic acid cycle for complete oxidation and decomposition, thereby releasing energy [60].

The function of GDH has also been studied in some parasites [25]. GDH of *Plasmodium falciparum* is expressed throughout the entire erythrocytic stage, and the NADPH generated by its catalytic reaction plays an important role in protecting *Plasmodium* from host oxidative damage and in maintaining survival [55]. Similarly, GDH of *Eimeria tenella* was found to be of great significance for its growth and development in host cells, and is involved in the process of spore invasion into host cells [59]. However, there are no relevant reports on *T. spiralis* GDH in the literature.

In this study, a *T. spiralis* GDH (TsGDH, GenBank: XM_003380129.1) was obtained from *T. spiralis* draft genome [34]. The aim of this study was to identify the biological characteristics and function of TsGDH in the metabolism, larval molting and development of *T. spiralis*.

## Materials and methods

### Ethics

The animal experiments in this study were carried out on the basis of National Guidelines for Experimental Animal Welfare of China. All animal experimental protocols were approved by the Zhengzhou University Life Science Ethics Committee (No. ZZUIRB GZR 2021-0044).

### Parasite, cell and animals

*Trichinella spiralis* isolate (ISS534) used in the research was collected from a naturally infected domestic pig (*Sus scrofa domesticus* Erxleben, 1777) in central China and passaged and maintained in BALB/c mice in our department. Human colon epithelial cell line Caco-2 was obtained from the Cell Resource Center of the Shanghai Institute for Biological Sciences of the Chinese Academy of Sciences [31]. Female BALB/c mice of six weeks of age were purchased from the Henan Provincial Experimental Animal Center (Zhengzhou, China).

### Collection of worms and protein preparation

At 42 days post infection (dpi), ML were collected from murine skeletal muscles after experimental infection with *T. spiralis* by the artificial digestion method [28]. The 6 and 24 h IIL, and 3 and 6 d AWs were recovered from the intestines of infected mice [30, 51]. The 6 d female AWs were cultivated in RPMI-1640 containing 10% fetal bovine serum at 37 °C and 5% CO_2_ for 24 h, and the NBL were collected from the culture medium [61, 74]. Soluble worm proteins and excretion-secretion (ES) proteins of various *T. spiralis* worm stages (ML, IIL, AW and NBL) were prepared as described before [17, 71].

### Bioinformatics analysis of TsGDH

The full-length cDNA sequence of the TsGDH gene is derived from GenBank (XM_003380129.1). Physical and chemical characteristics of the TsGDH were predicted with the ProtParam tool (https://web.expasy.org/protparam/). SignalP 5.0 (https://services.healthtech.dtu.dk/services/SignalP-5.0/) was served to predict TsGDH signal peptide. TMHMM-2.0 (https://services.healthtech.dtu.dk/services/TMHMM-2.0/) was used to predict the transmembrane region of TsGDH. Multiple alignments of TsGDH amino acid sequences with GDH from other *Trichinella* species/genotypes were performed using BioEdit software [66].The GenBank accession numbers of GDH from other *Trichinella* species and organisms were as follows: *Trichinella nativa* (KRZ50462.1), *Trichinella britovi* (KRY47484.1), *Trichinella pseudospiralis* (KRX88833.1), *Trichinella murrelli* (KRX38607.1), *Trichinella* T6 (KRX80455.1), *Trichinella nelsoni* (KRX20202.1), *Trichinella* T8 (KRZ84833.1) and *Trichinella patagoniensis* (KRY10565.1), *Trichinella* T9 (KRX66692.1), *Trichinella papuae* (KRZ71773.1), and *Trichinella zimbabwensis* (KRZ09175.1). The phylogenetic tree was constructed using MEGA 7.0 on the basis of the Neighbor-joining (NJ) method, as described before [15].

### Cloning, expression and purification of TsGDH

Total RNA was extracted from ML using Trizol (Invitrogen, Waltham, MA, USA) and reverse transcribed into cDNA, which was used as a template to amplify the TsGDH gene [72]. The complete length of the TsGDH cDNA sequence was amplified by PCR using specific primers carrying restriction enzyme sites *Bam*H I and *Sac* I **(bold**) (5′–TA**GGA TCC**ATGGCTTTGCGGCGGCTGTCT–3′, 5′–CGC**GAGCTC**TTAAGTAAAAGTAAA ACC GCCCAT–3′) [41]. The PCR products were cloned into the expression vector pQE-80L with a His-tag at the N-terminus (Novagen, Madison, WI, USA), and recombinant pQE-80L/TsGDH was introduced into *E. coli* BL21 (Novagen) [52]. The recombinant TsGDH (rTsGDH) was obtained by induction with 0.5 mM IPTG at 25 °C for 12 h. Subsequently, rTsGDH was purified by Ni–NTA His-tag affinity kit (Sangon Biotech, Shanghai, China) [13]. The identification of rTsGDH was performed by SDS-PAGE with 12% acrylamide separating gel as reported before [2, 62].

### Production of polyclonal antibodies against rTsGDH

Twenty mice were immunized by subcutaneous injection of 20 µg rTsGDH per mouse; the rTsGDH protein was pre-emulsified with equivalent volume of complete Freund’s adjuvant [73]. The mice received the same dose of rTsGDH mixed with incomplete Freund’s adjuvant at a 2-week interval for a total of 3 times [65]. Two weeks after the last immunization, tail blood of immunized mice was collected to isolate anti-rTsGDH immune serum sample. The antibody IgG titer of anti-rTsGDH serum was measured by ELISA with rTsGDH [1, 75].

### Western blot analysis

Bacterial proteins of pQE-80L/TsGDH/BL21 before and after induction, and purified rTsGDH were separated on 10% SDS-PAGE [49]. The proteins were transferred onto a nitrocellulose membrane (Millipore, Burlington, MA, USA) using the wet transfer cell (Bio-Rad, Hercules, CA, USA). The membrane was blocked with 5% skimmed milk in Tris-buffered saline containing 0.05% Tween (TBST) at 37 °C for 2 h, and cut into strips. The strips were probed by various sera (1:100; anti-rTsGDH serum, infection serum and pre-immune serum) at 37 °C for 2 h. After being washed with TBST, the strips were incubated at 37 °C for 1 h with HRP-anti-mouse IgG conjugate (1:10,000; Southern Biotech, Birmingham, AL, USA). After being washed again, the strips were colored using 3, 3'-diaminobenzidine tetrahydrochloride (DAB; Sigma-Aldrich, St. Louis, MO, USA) or an enhanced chemiluminescent kit (ECL, CWBIO, Taizhou, China) [23, 67].

### qPCR analysis of the TsGDH transcription level

Total RNA from various *T. spiralis* stages (ML, 6 h IIL, 3 and 6 d AWs, and NBL) was isolated using Trizol reagent (Invitrogen, USA), reverse-transcribed into cDNA using a PrimeScript™ RT reagent Kit (TaKaRa, Shiga, Japan) [22, 69]. A SYBR Green PCR master mix (TaKaRa) was used for qPCR in an ABI Prism 7500 Fast Sequence Detection System (Applied Biosystems, Foster City, CA, USA) [64]. The transcription level of TsGDH was assayed by using qPCR with specific primers (5′–GAAGATCACTCGTCGACTGGC–3′; 5′–ACGCATCCTTTTCGAGATGGC–3′). The specific primers for qPCR were designed using the specific primer design website (https://www.ncbi.nlm.nih.gov/tools/primer-blast/). It should be noted that at least one primer needs to be across the intron region, and the length of the amplified product is controlled between 100 bp and 300 bp [67]. To normalize the relative TsGDH transcription level, the transcription of a *T. spiralis* housekeeping gene GAPDH (GenBank AF452239) was subtracted. The 2^−ΔΔCt^ method was used to determine the relative transcription level of TsGDH, as previously described [16, 48].

### Indirect immunofluorescence test (IIFT)

IIFT was used to determine the expression and tissue localization of TsGDH in different *T. spiralis* stages [62, 66]. In brief, complete entire worms (ML, 6 and 12 h IIL, 3 and 6 d AWs, and NBL) were fixed with 4% paraformaldehyde at room temperature for 30 min and embedded in paraffin, and 2-µm-thick worm cross-sections were cut with a microtome, and then fixed in cold acetone at −20 °C for 20 min. The entire worms and cross-sections were probed with a 1:10 dilution of various sera (anti-rTsGDH serum, infection serum and pre-immune serum) at 37 °C for 2 h. After rinsing with phosphate-buffered saline (PBS), the worms and cross-sections were then incubated with Alex Fluor 488 conjugated with anti-mouse IgG conjugated (1:100; Abways, Shanghai, China). Following repeat washing, the immunofluorescence staining in worms and their cross-sections were examined under fluorescence microscopy (Olympus, Tokyo, Japan) [13, 15].

### RNA interference (RNAi)

Three pairs of TsGDH-specific dsRNA primers targeting the domain of the cDNA sequence of the TsGDH gene were designed (5′–GATCAC**TAATACGACTCACTATAGGG**GCGTCAAA TTCGTGGCATACT–3′, 5′–GATCAC**TAATACGACTCACTATAGGG**ATTTAATACACCT CGCCCAGT–3′; 5′–GATCAC**TAATACGACTCACTATAGGG**GGACTTGGCGGTATTCA TGGT–3′, 5′–GATCAC**TAATACGACTCACTATAGGG**ATTTTATCGGCAGCTGGTGTT–3′; 5′–GATCAC**TAATACGACTCACTATAGGG**AGGCTAAAATTGTTGCCGAAG–3′, 5′–G ATCAC**TAATACGACTCACTATAGGG**AAACCGCCCATCTGATATGAC–3′). The underlined letters are the enhancer sequence; the bold letters are the T7 promoter sequence. To confirm the specificity of TsGDH-dsRNA, the *T. spiralis* eukaryotic aspartyl protease (TsEasp, GenBank: XP_003373313.1) was used as a specific control. Furthermore, a green fluorescent protein (GFP)-dsRNA was also prepared as a negative control based on its sequence (5′–GATCAC**TAATACGACTCACTATAGGG**TCCTGGTCGAGCTGGACGG–3′, 5′–GATCAC**TAATACGACTCACTATAGGG**AAACCGCCCATCTGATATGAC–3′) [41]. Various kinds of dsRNAs were introduced into *T. spiralis* ML through electroporation and then incubated in RPMI 1640 medium at 37 °C for 1–3 days. The expression levels of TsGDH mRNA and protein were determined using qPCR and Western blotting, as described before [57]. GAPDH served as a reference gene for normalization. The expression level of TsGDH protein was evaluated by densitometry measurement using Image J software [68].

### Enzyme activity assay of natural TsGDH in worm somatic proteins

The enzyme activity of natural TsGDH in somatic soluble proteins from the dsRNA-treated ML was measured by UV spectrophotometry [55]. The enzyme activity reaction system contained 0.1 Mol/L PBS (pH 8.0), 10 mMol/L L-glutamic acid, and 500 μMol/L NAD^+^. The ML soluble proteins (2 μg) were added to the system (200 μL) and incubated at 37 °C for 30 min. Finally, the absorbance change in the reaction system was monitored at 340 nm [45].

### Effects of dsRNA-TsGDH on *in vitro* larval metabolism

A total of 3,000 ML were transfected with 50 ng/μL dsRNA-TsGDH, dsRNA-GFP and PBS, and cultured for 2 days. The ammonia nitrogen excretion of ML in culture medium was assayed by the Nessler reagent method [35, 78]. Larval ATP contents from different groups were measured by ATP assay kit (Sangon Biotech, Shanghai, China). Distribution of glycogen and lipid droplets in ML was observed by PAS and oil red O staining [14, 40]. Furthermore, the larval soluble somatic proteins were prepared and total sugar and lipid content in somatic soluble proteins were measured as previously described [70].

### The *in vitro* larval invasion and molting assay

In order to investigate the effect of dsRNA-TsGDH on *in vitro* larval invasion of the intestinal epithelium, *in vitro* larva invasion of Caco-2 cell monolayer was performed [29, 74]. Briefly, Caco-2 cells were cultured in a 24-well cell culture plate until the confluence. The ML treated with dsRNA-TsGDH was activated into the IIL with 5% swine bile at 37 °C for 2 h. In a 24-well plate, 50 IIL were added onto the surface of Caco-2 monolayer in Dulbecco’s Modified Eagle Medium (DMEM) semisolid medium (DMEM supplemented with L-glutamine, 15 mM HEPES, and 1.75% agarose). After cultivation for 2 h, the larvae were observed under a light microscope. The invaded larvae were active and migrated to the monolayer and damaged the cell monolayer, whereas the non-invaded larvae were coiled on the surface of cells and the monolayer was intact [23, 31]. To investigate the inhibition of dsRNA-TsGDH on larval molting, the larvae cultured for 3 d were examined and counted under light microscope [41, 70].

### Challenge of mice with the ML transfected by dsRNA-TsGDH

To further validate the role of TsGDH in *T. spiralis* metabolism and growth, 120 mice were randomly divided into three groups (40 mice in each group). The ML were transfected with 50 ng/μL dsRNA-TsGDH and cultured for 2 days, and then each mouse was orally inoculated with 200 ML. At 24 h, 3 and 6 d, and 35 dpi, 10 mice of each group were euthanized, and the IIL, AWs and ML were collected and counted, respectively, and their morphology and size were observed and measured [12]. Moreover, female adult fecundity (reproductive ability) was evaluated by measuring the number of NBL/each female within a 72 h period, and the length of NBL was measured [66]. TsGDH enzyme activity, carbohydrate and lipid content in worms, and the excretion yield of ammonia nitrogen were measured, as previously reported [22, 79].

### Statistical analysis

The obtained data were analyzed using SPSS 21.0 software and are shown as arithmetic mean ± SD (standard deviation). One-way ANOVA was used to analyze the difference of TsGDH mRNA and protein expression, enzyme activity, content of ATP, sugar and lipids, ammonia nitrogen excretion, and worm burden and length. The differences in larval molting and invasion rates among various groups were analyzed using a Chi-square test. The statistical difference level was *p < *0.05.

## Results

### Bioinformatics analysis of TsGDH

**Figure 1:**
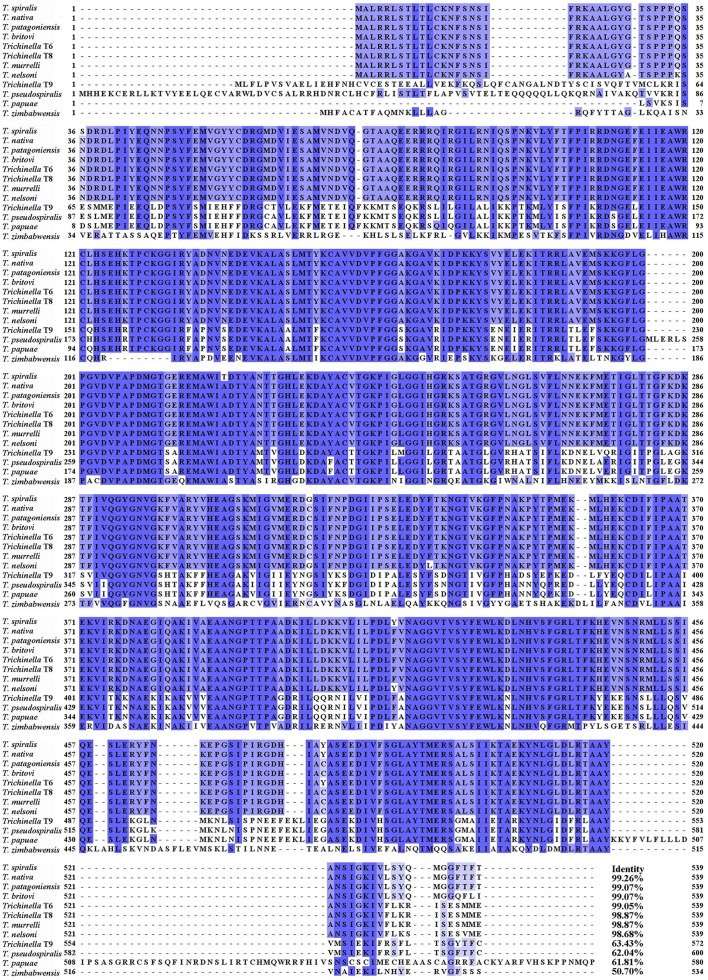
Multisequence alignment of glutamate dehydrogenase of different species/genes of *Trichinella*. According to the analysis of Cluster Omega, the same amino acids are marked in deep blue and conservative substitution of amino acid residues are marked in light blue. The glutamate dehydrogenase gene of different species/genotypes of *Trichinella* has high homology. The number at the end of each sequence represents the percentage of identity with TsGDH.

**Figure 2:**
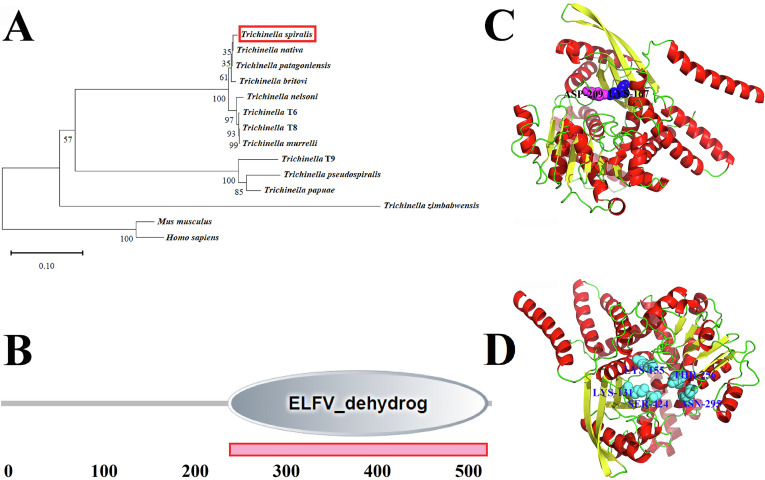
Phylogenetic tree construction, prediction of function domain, and tertiary structure of TsGDH. A: TsGDH in the evolutionary tree of *Trichinella*, humans and the mouse. The evolutionary tree of glutamate dehydrogenase of 12 different species/genotypes of the genus *Trichinella* was constructed by the neighbor-joining (NJ) method. B: TsGDH has an ELFV_dehydrog domain at 249-535 aa. C: Prediction of the tertiary structure of TsGDH. The homologous modeling and prediction of the tertiary structure of TsGDH were achieved using Alphafold2. TsGDH has one enzyme active site (Lys 167) displayed as a dark blue spherical shape, and one catalytic site (Asp 209) displayed as a purple spherical shape. D: The substrate binding sites of TsGDH are displayed in a light blue spherical shape, including Lys 131, Lys 155, Thr 256, Asn 295, and Ser 424.

The complete length cDNA sequence of TsGDH is 1,620 bp and encodes 539 amino acids. The predicted molecular weight of TsGDH protein is 59.74 kDa, with an isoelectric point (pI) of 8.24. TsGDH has no transmembrane region and no signal peptide, but has a distinct hydrophobic structure at the N-terminus. The subcellular location of TsGDH is localized on the mitochondrial membrane. The amino acid sequence of TsGDH exhibited a similarity of 99.26, 99.07, 99.07, 99.05, 98.87, 98.87, 98.68, 63.43, 62.04, 61.81, and 50.70% with glutamate dehydrogenase from *T. nativa*, *T. patagoniensis*, *T. britovi*, *Trichinella* T6, *Trichinella* T8, *T. murrelli, T. nelsoni,* *Trichinella* T9, *T. pseudospiralis,* *T. papuae* and *T. zimbabwensis* (Fig. 1). The phylogenetic tree exhibited the monophyletic cluster of the 12 *Trichinella* species/genes. TsGDH exhibited a more intimate evolutionary relationship among the 9 encapsulated species of the *Trichinella* genus (Fig. 2A). It has an ELFV_dehydrogenase domain at 249–535 aa predicted on SMART (Fig. 2B). The Alphafold2 structure prediction showed that TsGDH had 18 helixes (red) and 11 sheets (yellow). Also, TsGDH has one enzyme active site (Lys 167) and one catalytic site (Asp 209), and five substrate binding sites (Lys 131, Lys 155, Thr 256, Asn 295, and Ser 424) (Figs. 2C, 2D).

### Expression and identification of TsGDH

**Figure 3:**
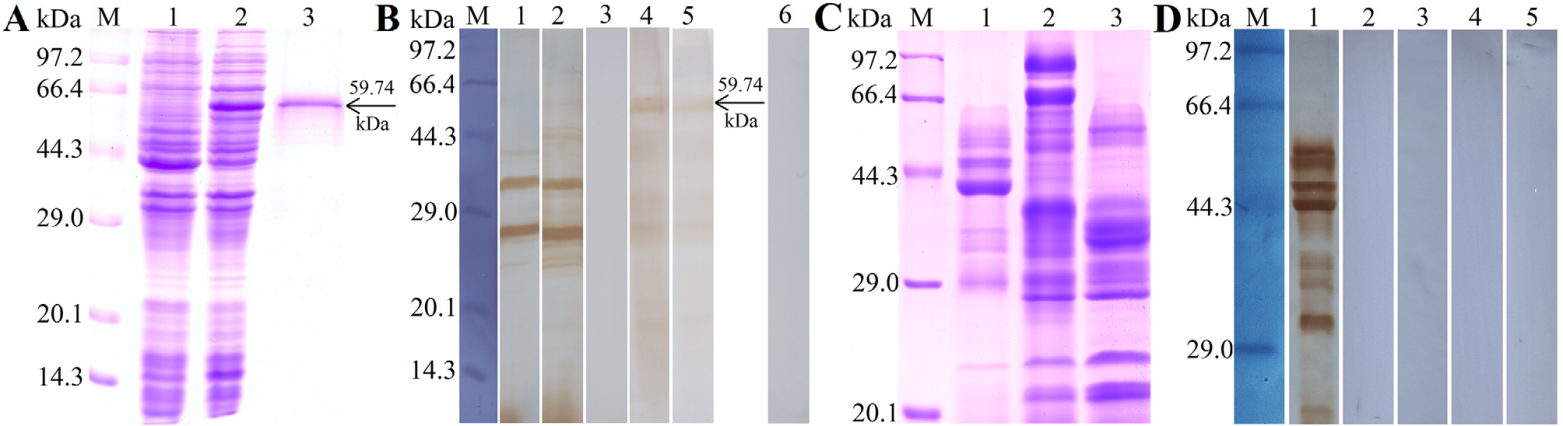
Expression and identification of rTsGDH. A: SDS-PAGE analysis of rTsGDH. Lane M: protein marker; Lane 1: BL21 carrying pQE-80L/TsGDH before induction. Lane 2: BL21 carrying pQE-80L/TsGDH after being induced with 0.5 mM IPTG at 25 °C for 12 h. Lane 3: purified rTsGDH indicated by a black arrow. B: Western blotting analysis of rTsGDH antigenicity. Lane 1: BL21 carrying pQE-80L/TsGDH before induction was not recognized by infection serum. Lane 2: BL21 carrying pQE-80L/TsGDH after induction was not recognized by infection serum. The purified rTsGDH was recognized by anti-rTsGDH serum (Lane 4) and anti-his tag McAb (Lane 5), but not by infection serum (Lane 3) and normal serum (Lane 6). C: SDS-PAGE analysis of ES proteins of *T. spiralis* ML (Lane 1), IIL (Lane 2) and 6 d AW (Lane 3). D: Western blotting analysis of *T. spiralis* worm ES proteins. The ML ES proteins were recognized by infection serum (Lane 1), but not by anti-rTsGDH serum (Lane 2) and normal serum (Lane 3). The ES proteins of IIL (Lane 4) and 6 d AW (Lane 5) were not recognized by anti-rTsGDH serum.

The SDS-PAGE results revealed that the molecular weight (MW) of the fusion protein rTsGDH expressed by the BL21 bacteria carrying pQE-80L/TsGDH was 59.74 kDa, which was consistent with the predicted MW of TsGDH (Fig. 3A). In order to evaluate the IgG antibody response elicited by rTsGDH immunization, the titer of anti-rTsGDH IgG at two weeks after final immunization was measured by ELISA and the results showed that the IgG titer of anti-rTsGDH antibodies reached 1:10^5^, indicating that rTsGDH has good antigenicity. Western blot results showed that rTsGDH was recognized by anti-rTsGDH serum and anti-his tag monoclonal antibody (McAb), but not by infection serum and pre-immune serum (Fig. 3B). Moreover, the ES proteins of ML, IIL and 6 d AWs were not identified by anti-rTsGDH serum, suggesting that TsGDH is a worm somatic protein, but not a secretory protein of this nematode (Figs. 3C, 3D).

### Transcription and expression of TsGDH gene in diverse *T. spiralis* stages

**Figure 4:**
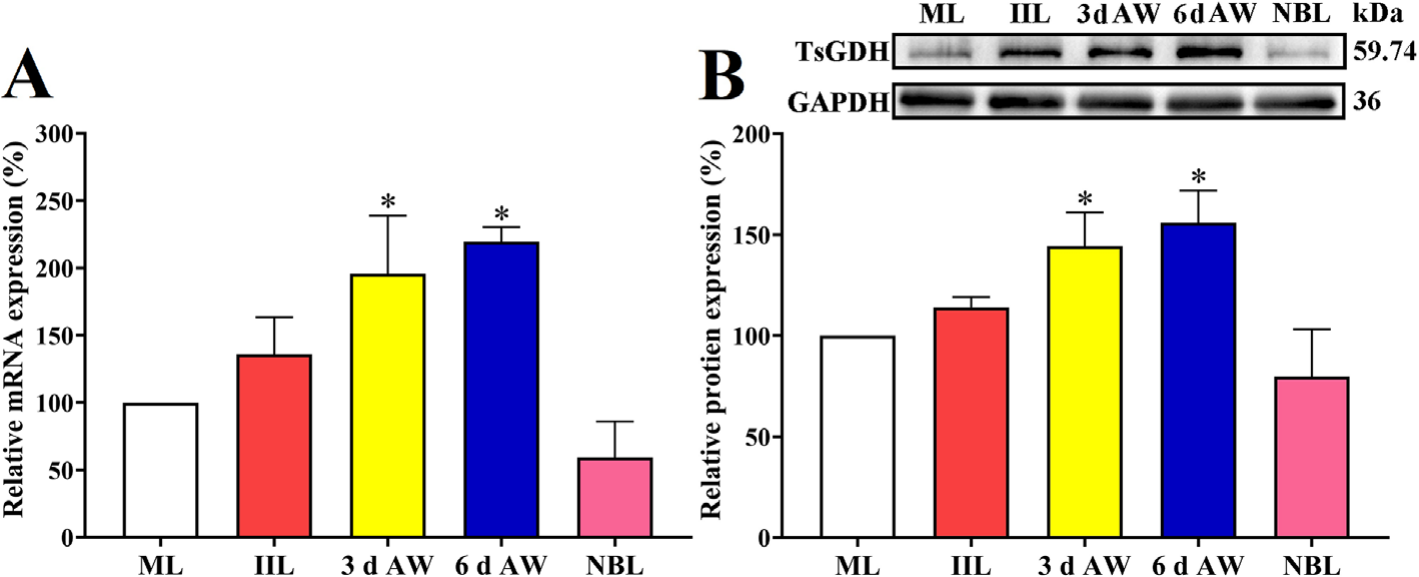
Quantification of TsGDH expression in diverse *Trichinella spiralis* stages performed by qPCR and Western blotting. A: qPCR assay of TsGDH mRNA expression in diverse *T. spiralis* stages. The TsGDH transcription level was assessed by the 2^− ΔΔCt^ method. GAPDH served as an internal control. B: Western blot analysis was performed to determine the TsGDH protein expression level in various *T. spiralis* stages. Transcription and expression levels of TsGDH at 3 and 6 d AWs was obviously higher than other worm stages. *p < 0.01 compared with the ML stage.

The qPCR results revealed that transcription levels of TsGDH in 3 d and 6 d AW stages were distinctly higher than the ML stage (*F = *13.51, *p < *0.01) (Fig. 4A). Furthermore, the expression level of TsGDH protein in 3 d and 6 d AW stages was also obviously higher than the ML stage (*F = *13.28，*p < *0.001) (Fig. 4B). The findings showed that TsGDH was expressed in all phases of *T. spiralis*, with a higher expression level in the AW stage, suggesting that TsGDH is an adult-highly expressed GDH gene, which might be involved in worm reproduction and development.

### Expression and localization of natural TsGDH in diverse *Trichinella spiralis* stages

**Figure 5:**
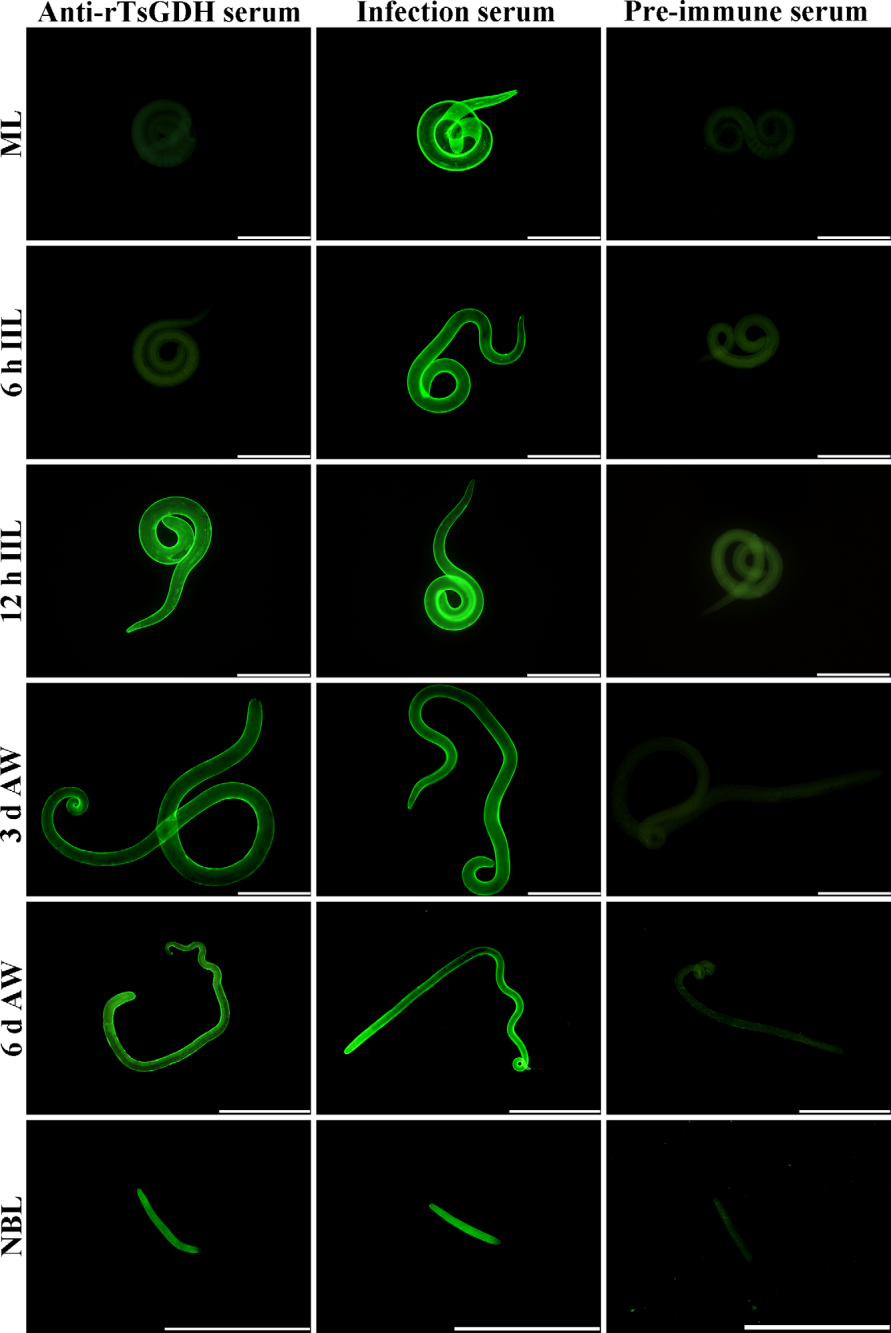
IIFT detection of TsGDH in the epicuticle of different *Trichinella spiralis* stages. IIFT was conducted to detect the TsGDH expression on epicuticle of *T. spiralis* using diverse sera (anti-rTsGDH serum, infection serum, and pre-immune serum). Bright green fluorescence was observed on the outer cuticle of 12 IIL, 3 and 6 d AWs and NBL, but not in ML and 6 h IIL stages. Infection serum and pre-immune serum served as positive and negative controls. Scale bars for ML, 6 h, 12 h IIL, 3 d AWs and NBL: 200 μm; scale bar for 6 d AWs: 500 μm.

**Figure 6:**
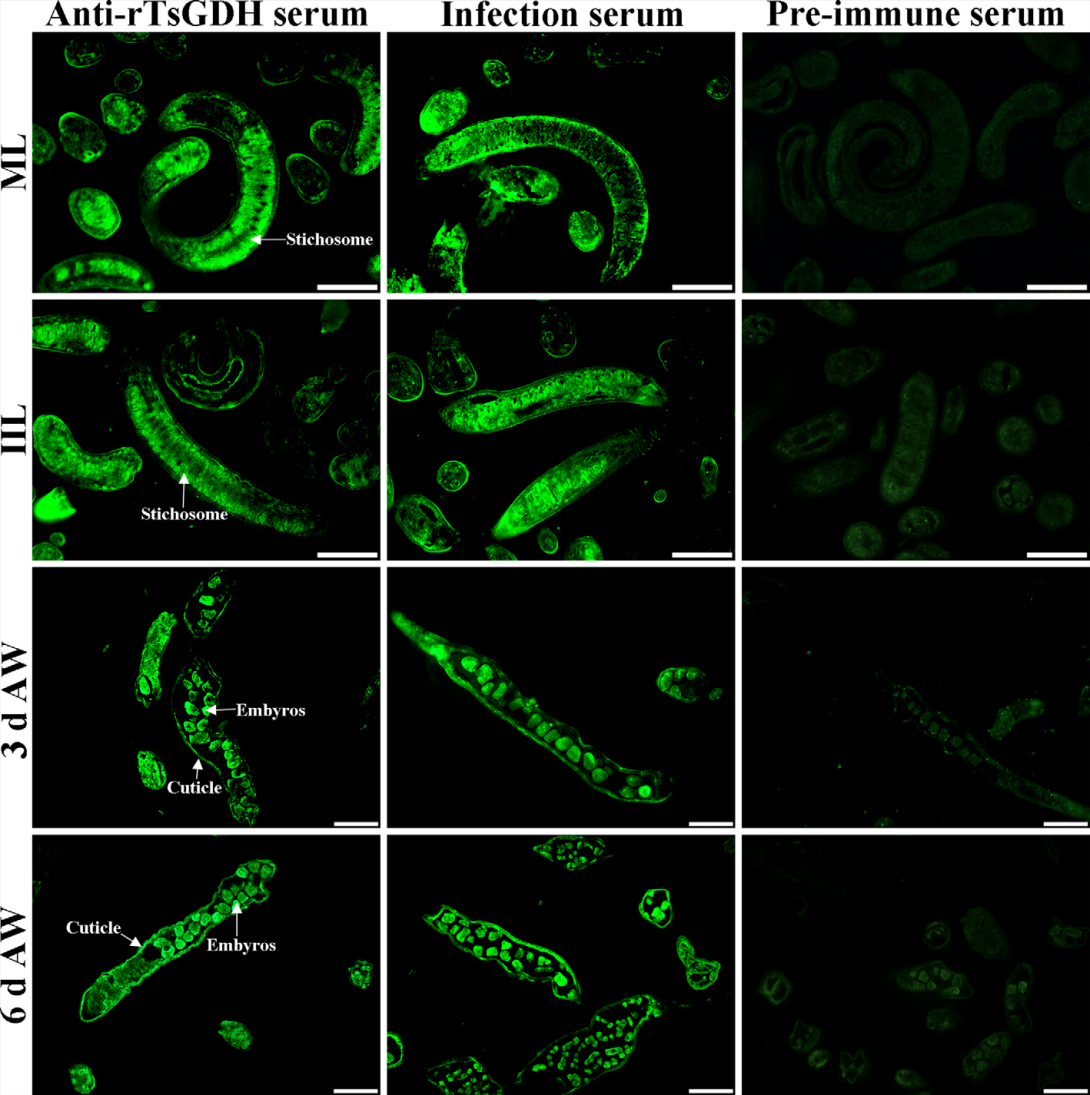
Immunofluorescent localization of TsGDH in cross-sections of *Trichinella spiralis* worms by IIFT. IIFT with immune serum was employed to identify the expression and localization of TsGDH in diverse *T. spiralis* stages. Immune fluorescent staining was detected on cuticle, muscle and stichosome, and intrauterine embryos of female adults by anti-rTsGDH serum. Pre-immune serum as negative control did not detect any immunostaining in worm cross-sections. Scale bars: 100 μm.

The results of IIFT with whole parasites showed that anti-rTsGDH serum recognized the outer epidermis of various worm stages (12 h IIL, 3 and 6 d AW, and NBL), except for the ML and 6 h IIL stage (Fig. 5). Furthermore, the IIFT with cross-sections revealed that TsGDH was mainly localized in the cuticle, muscular layer and stichosome of ML, IIL and AWs, and around embryos of female adults (Fig. 6).

### Silencing the TsGDH gene decreased TsGDH expression and enzymatic activity

**Figure 7:**
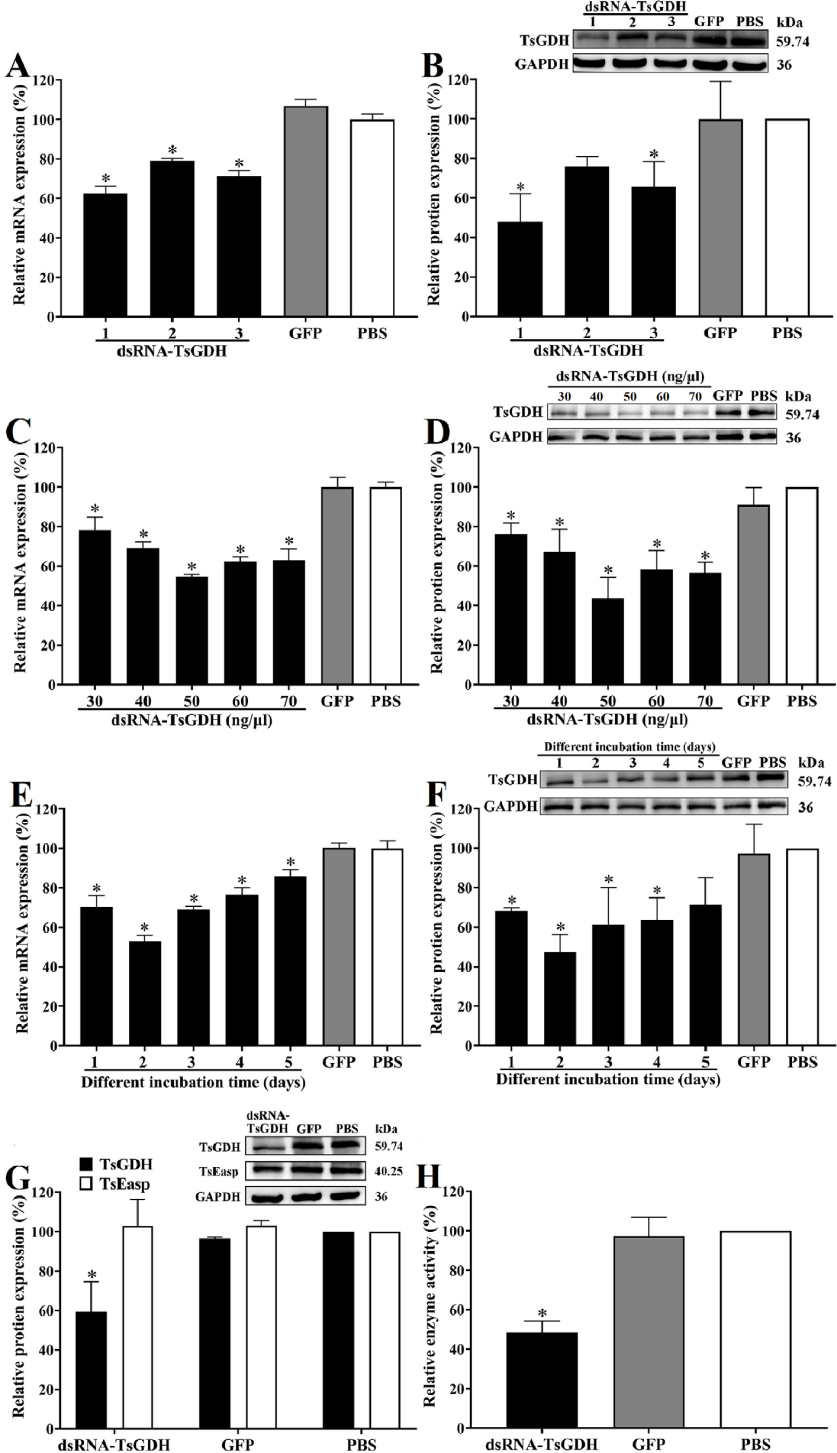
Silencing TsGDH gene suppressing TsGDH expression and enzymatic activity. A: TsGDH transcription levels in ML transfected with different dsRNA-TsGDH. B: TsGDH expression levels in ML transfected with different dsRNA-TsGDH. C: TsGDH transcription levels in ML transfected with various doses of dsRNA-TsGDH. D: TsGDH expression levels in ML transfected with various doses of dsRNA-TsGDH. E: TsGDH transcription levels in ML at 1–5 days after transfection with 50 ng/μL dsRNA-TsGDH. F: TsGDH expression levels in ML at 1–5 days after transfection with 50 ng/μL dsRNA-TsGDH. G: Expression levels of TsGDH and TsEasp in ML treated with dsRNA-TsGDH. H: TsGDH enzyme activity in ML treated with dsRNA-TsGDH. *p < 0.05 relative to the PBS group.

Following the introduction of 30 ng/μL dsRNA-TsGDH 1-3 and a 2-day incubation, the survival rates of the larvae transfected with dsRNA-TsGDH 1-3, dsRNA-GFP, and PBS were 90.59, 90.70, 90.58, 90.62, and 90.60%, respectively (*F = *0.074, *p > *0.05). These results indicate that electroporation did not significantly impact larval survival. After the ML was treated with 30 ng/μL dsRNA-TsGDH 1-3 and cultured for 2 days, the TsGDH transcriptional level was decreased by 37.37%, 21.24%, and 28.84%, respectively, compared with the PBS group (*F = *125.4, *p < *0.0001). Additionally, the TsGDH expression level in the dsRNA-TsGDH 1-3 group was decreased by 52.09%, 23.88%, and 34.33%, respectively (*F = *10.05, *p < *0.05) (Figs. 7A, 7B). Hence, dsRNA-TsGDH 1 was used in the following study. After transfection with different concentrations of dsRNA-TsGDH (30, 40, 50, 60, and 70 ng/μL) and cultured for 2 days, compared with the PBS group, the TsGDH transcription level was decreased by 21.92%, 30.68%, 45.36%, 37.67%, and 36.92% (*F = *55.93, *p < *0.0001), and TsGDH expression level was decreased by 23.60%, 32.67%, 56.50%, 41.63%, and 43.31%, respectively (*F = *18.06, *p < *0.0001) (Figs. 7C, 7D), indicating that 50 ng/μL dsRNA is the optimal transfection dose. When the ML were treated with 50 ng/μL dsRNA-TsGDH and cultured for 1–5 days, the TsGDH transcription level was decreased by 29.67%, 46.97%, 31.21%, 23.51%, and 14.17% (*F = *74.61, *p < *0.0001), and TsGDH expression levels were decreased by 31.81%, 52.77%, 38.70%, 36.33%, and 28.64%, respectively (*F = *8.004, *p < *0.001) (Figs. 7E, 7F). Moreover, the TsEasp protein expression level was not obviously suppressed in ML treated with dsRNA-TsGDH, suggesting that dsRNA-TsGDH specifically targets TsGDH (Fig. 7G). Therefore, the larvae treated with 50 ng/μL dsRNA-TsGDH and cultured for 2 days were utilized for the subsequent experiment. The enzymatic activity assay showed that natural TsGDH enzyme activity in soluble proteins of the ML treated with dsRNA-TsGDH was decreased by 51.67%, compared with the PBS group (*F = *57.81, *p < *0.001) (Fig. 7H). The results demonstrated that RNAi significantly decreased TsGDH expression and enzymatic activity.

### Suppression of dsRNA-TsGDH on larval metabolism

**Figure 8:**
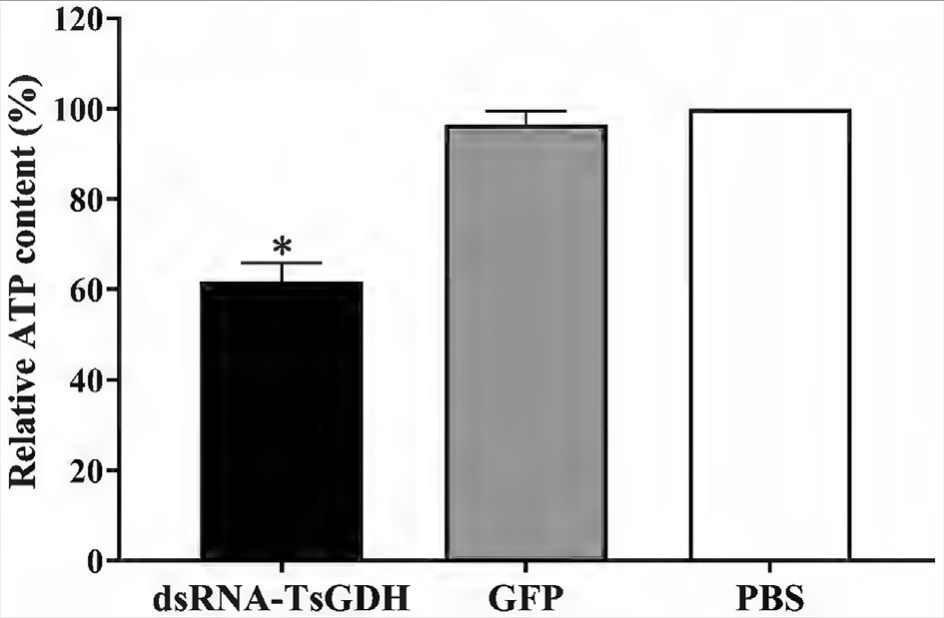
Effect of suppression of dsRNA-TsGDH on larval ATP content. *p < 0.05 relative to the PBS group.

**Figure 9:**
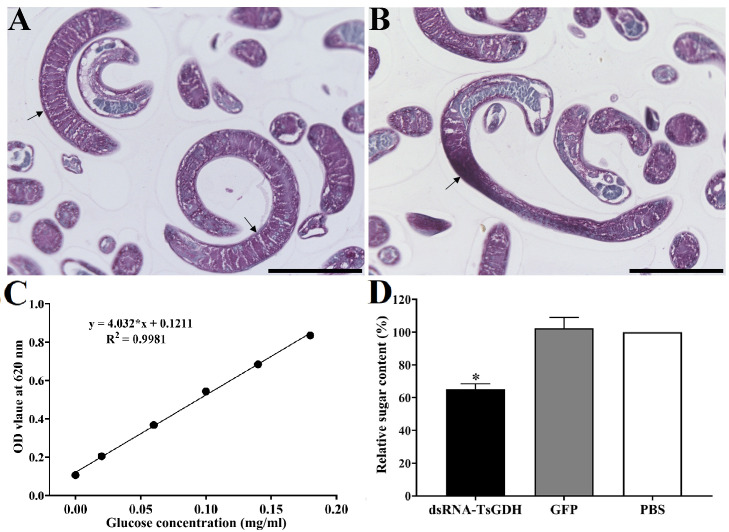
Effect of suppression of dsRNA-TsGDH on larval sugar content. Glycogen is mainly distributed in stichosome, muscular layer (A) and around the intestine of the ML (B). C: Sugar standard curve. D: dsRNA-TsGDH reduced larval total sugar content. The arrows indicate glycogen. *p < 0.05 relative to the PBS group. Scale bars: 50 μm.

**Figure 10:**
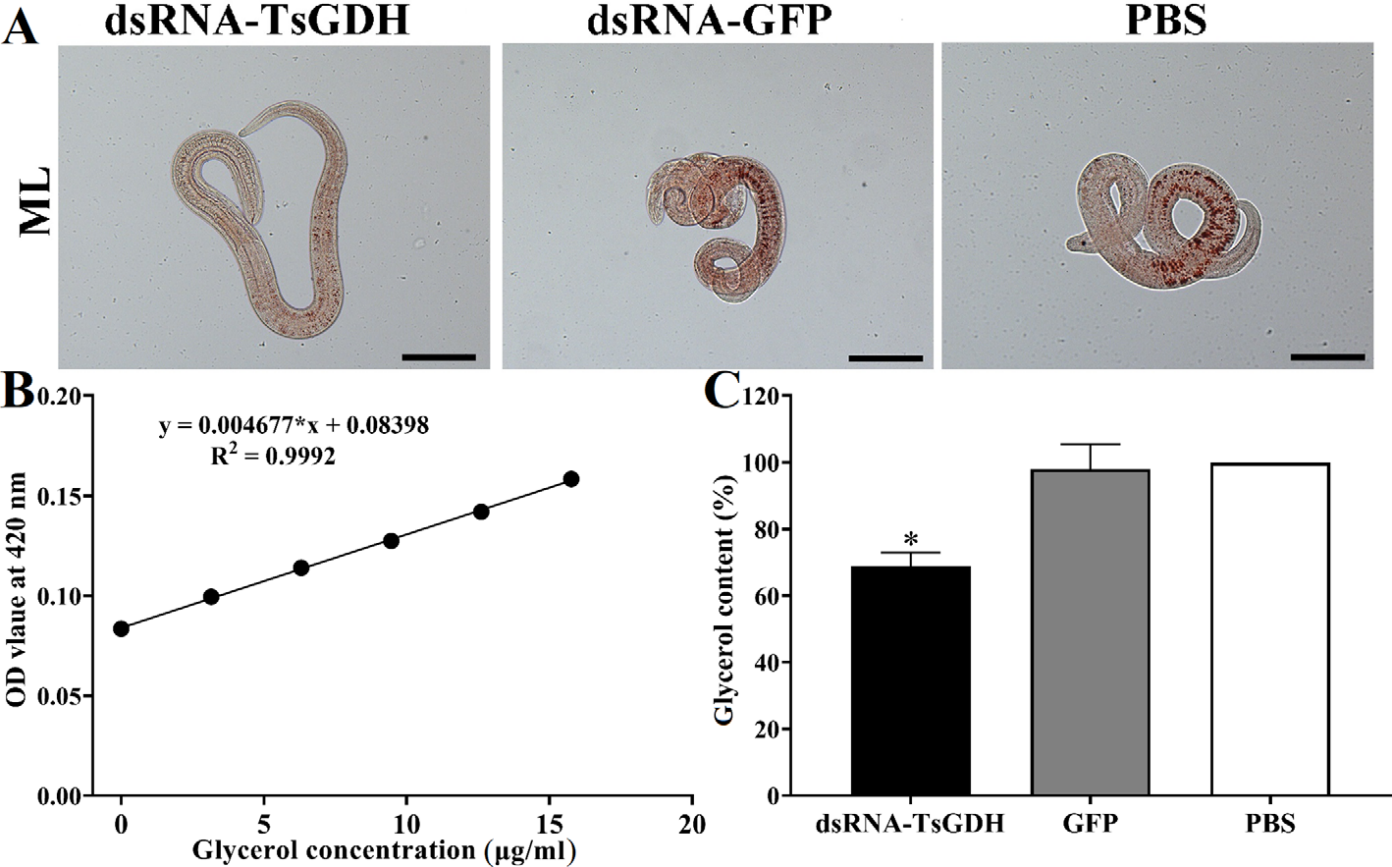
Effect of suppression of dsRNA-TsGDH on larval lipid content. A: Distribution of lipid droplets in different groups of *T. spiralis* muscle larvae. The complete lipid composition of the entire larva are dyed brown by oil red O, and smaller lipid droplets were evenly distributed in muscle larvae. The stichosome of ML had larger lipid droplets, which were reddish brown in color. The darker the color, the higher the lipid content. B: Glycerol standard curve. C: dsRNA-TsGDH reduced larval total lipid content. *p < 0.05 relative to the PBS group. Scale bars: 100 μm.

**Figure 11:**
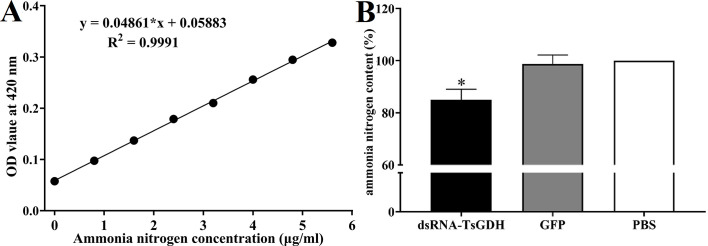
Effect of suppression of dsRNA-TsGDH on larval ammonia nitrogen production. A: Ammonia nitrogen concentration standard curve. B: dsRNA-TsGDH reduced larval ammonia nitrogen production. *p < 0.05 relative to the PBS group.

After treatment with dsRNA-TsGDH, the ATP content in ML of the dsRNA-TsGDH, GFP, and PBS groups was 4.9098 × 10^−2^, 7.6964 × 10^−2^, and 7.9618 × 10^−2^ μMol, respectively. Compared with the PBS group, the ATP content in ML treated with dsRNA-TsGDH was decreased by 38.23% (*F = *156.9, *p < *0.0001) (Fig. 8). The PAS staining results showed that the glycogen in ML was distributed in the stichosome, muscular layer, and around the gut. Compared with the PBS group, the glycogen content of the dsRNA-TsGDH group had a 34.84% reduction (*F = *72.58, *p < *0.001) (Fig. 9). Also, the results of oil red O staining showed that the lipids of the entire larvae were dyed brown, and smaller lipid droplets were distributed in whole muscle larvae. The stichosome of ML had larger lipid droplets, which were reddish brown in color. After treatment with dsRNA-TsGDH, the lipid content of ML was decreased by 31.02% (*F = *38.41, *p<* 0.001) (Fig. 10). In addition, ammonia nitrogen production of ML from the dsRNA-TsGDH group had a 14.98% decrease, compared with the PBS group (*F = *22.78, *p < *0.01) (Fig. 11).

### Inhibition of dsRNA-TsGDH on larval invasion and molting *in vitro*

**Figure 12:**
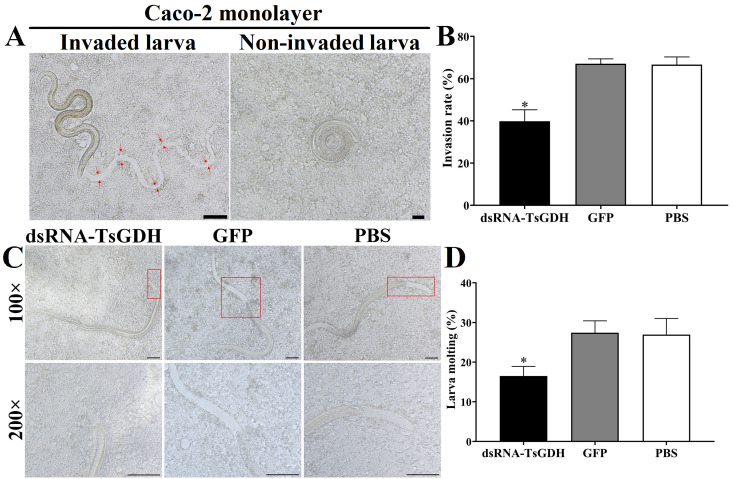
dsRNA-TsGDH inhibition of *Trichinella spiralis* larval invasion and molting *in vitro*. A: *T. spiralis* larva damage and invasion into the Caco-2 cell monolayer observed under a microscope. The migratory larva extended and migrated in the Caco-2 monolayer. The red arrow shows the larval migratory trace into the Caco-2 monolayer. The non-invaded larva was coiled. Scale bar: 100 μm. B: Inhibitory effect of dsRNA-TsGDH on *T. spiralis* larva invasion. C: Larval molting in different groups. No obvious molting traces in larval tail were observed in the dsRNA-TsGDH group, while obvious molting sheaths in the larval head and tail were observed in the GFP and PBS groups. D: dsRNA-TsGDH reduced the molting rate of the larvae. The area in the red box was enlarged for observation. *p < 0.05 relative to the PBS group.

The results of the *in vitro* larval invasion assay showed that dsRNA-TsGDH obviously impeded larval invasion and molting. After treatment with dsRNA-TsGDH, the ability of larvae to invade Caco-2 cells was distinctly weakened. Compared with the PBS group, the larval invasion of the dsRNA-TsGDH group was reduced by 40.21% (*F = *45.78, *p < *0.001) (Figs. 12A, 12B). Furthermore, the larval molting of the dsRNA-TsGDH group was inhibited by 38.73% (*F = *10.89, *p < *0.05) (Figs. 12C, 12D).

### dsRNA-TsGDH inhibited larval infectivity, development, and female fecundity

**Figure 13:**
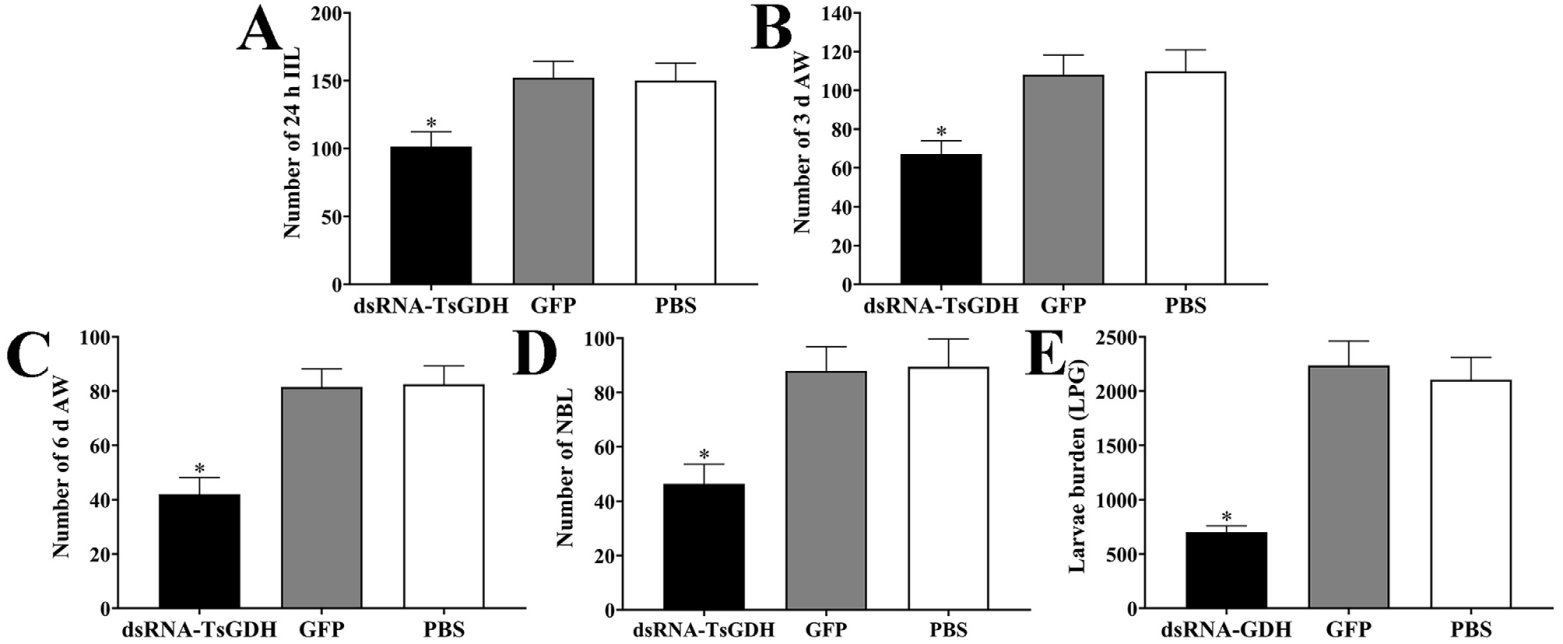
dsRNA-TsGDH reduction of *Trichinella spiralis* burden and female reproductive capacity in infected mice. A: Number of the 24 h IIL. B: Number of 3 d AWs. C: Number of 6 d AWs. D: NBL production of 6 d female worms cultured *in vitro* for 72 h. E: Muscle larva burden (larval burden per gram of muscle, LPG) at 35 days post infection. *p < 0.05 relative to the PBS group.

Compared with the PBS group, the worm burdens of IIL, 3 and 6 d AWs, and ML of dsRNA-TsGDH group were reduced by 32.49%, 38.80%, 48.97%, and 66.75% (*F*_ IIL_ *= *57.61, *F*_3 d AW_ *= *70.16, *F*_6 d AW_ *= *125.5, *F*_ML_ *= *224.7; *p < *0.0001). NBL production per female in 72 h of the dsRNA-TsGDH group was reduced by 43.20% (*F = *75.42, *p < *0.0001) (Fig. 13), demonstrating that dsRNA-TsGDH distinctly inhibited larval infectivity, development, and female production ability.

**Figure 14:**
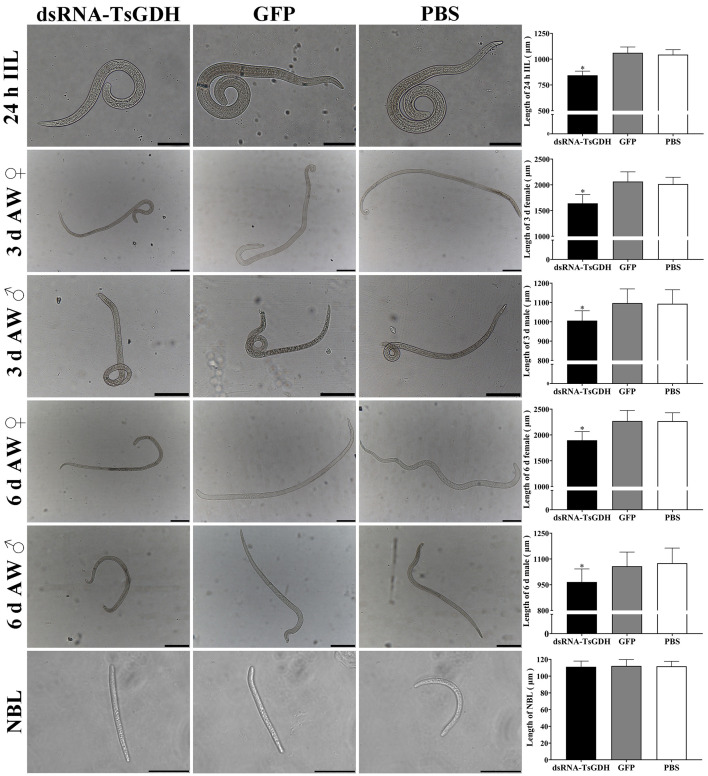
dsRNA-TsGDH inhibition of *Trichinella spiralis* development in infected mice. After RNAi treatment, the length of *T. spiralis* IIL, and 3 and 6 d female and male AWs from infected mice was significantly shortened. Scale bar: 200 μm for 24 h IIL, 3 d and 6 d AWs; 50 μm for NBL. *p < 0.05 relative to the PBS group.

In addition, the morphology of diverse stage worms from various group was observed and photographed under a microscope. Compared with the PBS group, the length of 24 h IIL, 3 d females and males, 6 d females and males from the dsRNA-TsGDH group was decreased by 19.19%, 18.54%, 7.99%, 16.26%, and 10.16% (*F*_24 h IIL_ *= *185.7, *F*_3 d female_ *= *58.93, *F*_3 d male_ *= *18.09, *F*_6 d female_ *= *42.78, *F*_6 d male_ *= *15.18; *p < *0.0001), respectively (Fig. 14). However, there was no significant difference in the NBL length of various groups (*p >* 0.05). These findings suggested that dsRNA-TsGDH evidently impeded larval growth, but did not affect the next generation NBL.

### Inhibition of dsRNA-TsGDH on metabolism and natural TsGDH enzyme activity of *Trichinella spiralis* in infected mice

**Figure 15:**
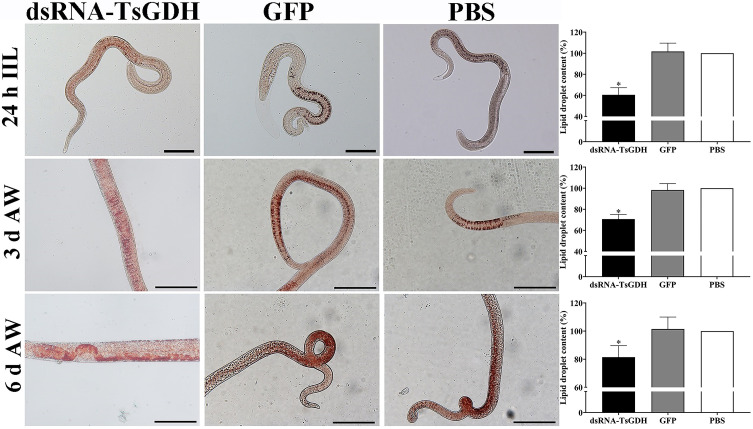
Oil red O staining of various *Trichinella spiralis* stages from infected mice challenged with dsRNA-TsGDH treated ML. Lipid droplets were mainly distributed in the IIL stichosome, adult worm intestines and female ovaries. The color of lipid droplets in the dsRNA-TsGDH group was lighter, and there were fewer large lipid droplets, and the lipid content of the dsRNA-TsGDH group was clearly reduced, compared to the PBS group. Scale bar: 100 μm. *p < 0.05 relative to the PBS group.

The results of oil red O staining showed that lipid droplets were mainly distributed in the stichosome of IIL and AWs, as well as in the intestines and ovaries of females (Fig. 15). Compared with the PBS group, lipid droplet content in IIL, 3 and 6 d AWs of the dsRNA-TsGDH group was reduced by 39.45%, 29.28%, and 18.52%, respectively (*F*_IIL_ *= *45.33, *p < *0.001; *F*_3 d AW_ *= *43.11, *p < *0.001; *F*_6 d AW_ *= *8.046, *p < *0.05).

**Figure 16:**
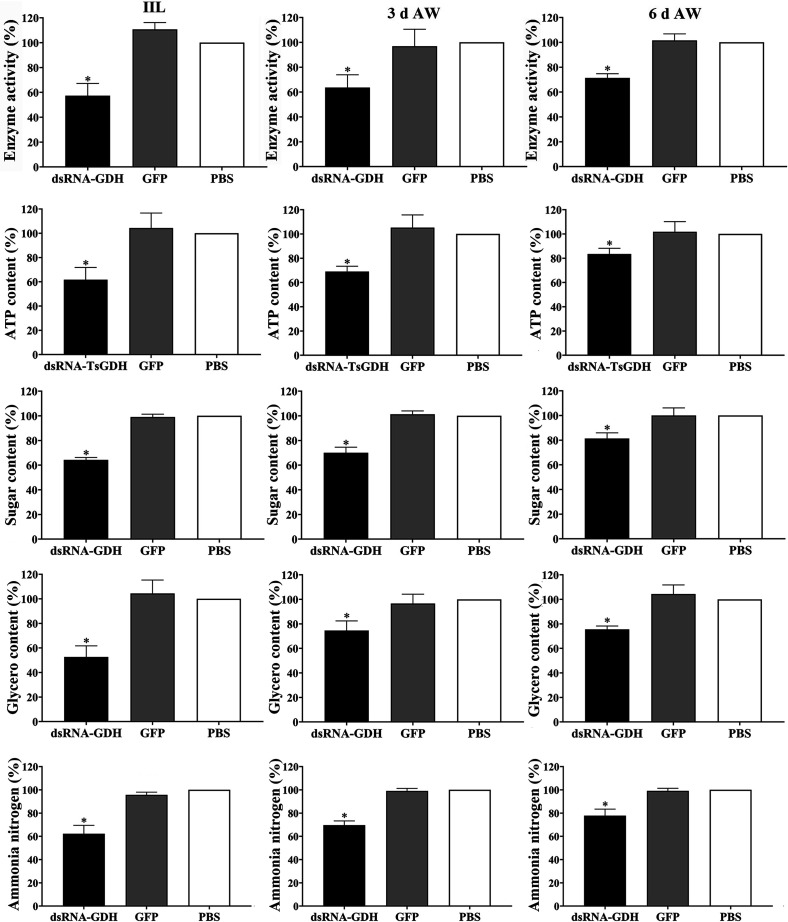
RNAi inhibition of natural TsGDH enzyme activity and metabolism of intestinal *Trichinella spiralis* worms from infected mice. The natural TsGDH enzyme activity, content of ATP, sugar and lipids, and ammonia nitrogen excretion of 24 h IIL, 3 and 6 d AWs were significantly reduced in the dsRNA-TsGDH group. *p < 0.05 relative to the PBS group.

The enzyme activity assay results showed that compared with the PBS group, the enzyme activity of 24 h IIL, and 3 and 6 d AWs from the dsRNA-TsGDH group was decreased by 42.65%, 36.39%, and 28.66%, respectively (*F*_24 h IIL_ *= *56.46, *p < *0.001; *F*_3 d AW_ *= *12.63, *p < *0.01; *F*_6 d AW_ *= *65.49, *p < *0.001). In the dsRNA-TsGDH group, the ATP content of 24 h IIL, and 3 and 6 d AWs was decreased by 38.30%, 30.96%, and 16.54%, respectively (*F*_24 h IIL_ *= *19.45, *F*_3 d AW_ *= *26.45, *p < *0.01; *F*_6 d AW_ *= *9.741, *p < *0.05); the sugar content was decreased by 35.77%, 29.93%, and 18.63%, respectively (*F*_24 h IIL_ *= *393.9, *F*_3 d AW_ *= *101.5, *p < *0.0001; *F*_6 d AW_ *= *17.74, *p < *0.01); the lipid content in three stage worms was decreased by 47.41%, 25.41%, and 24.42% (*F*_24 h IIL_ *= *36.62, *F*_3 d AW_ *= *14.40, *p < *0.01; *F*_6 d AW_ *= *34.84, *p < *0.05); and the ammonia nitrogen excretion was decreased by 37.76%, 30.33%, and 22.24%, respectively (*F*_24 h IIL_ *= *66.23, *F*_3 d AW_ *= *151.6, *F*_6 d AW_ *= *39.37, *p < *0.001) (Fig. 16). The results demonstrated that RNAi distinctly suppressed the sugar, lipid, and protein metabolism of intestinal *T. spiralis* worms (IIL and adults).

### Inhibition of dsRNA-TsGDH on the IIL molting in infected mice

**Figure 17:**
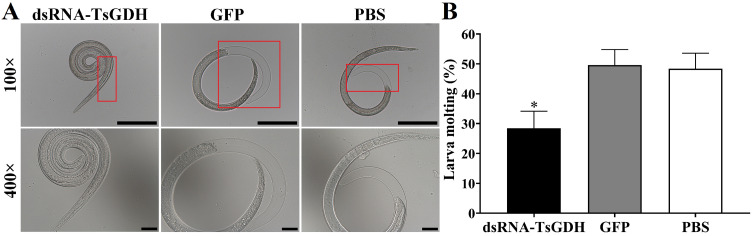
dsRNA-TsGDH inhibition of IIL molting in infected mice. A: The sheath of the molting IIL larvae in the GFP and PBS groups could be clearly observed, while the IIL sheath in the dsRNA-TsGDH group was invisible. The area in the red box was enlarged for observation. Scale bar: 100× is 200 μm. 400× is 50 μm. B: dsRNA-TsGDH reduced *in vivo* IIL molting. *p < 0.05 relative to the PBS group.

The results of the challenge experiment showed that dsRNA-TsGDH could significantly inhibit IIL molting. The molting rates of 24 h IIL in the dsRNA-TsGDH, GFP, and PBS groups were 28.4%, 49.6%, and 48.4%, respectively. Compared with the PBS group, the IIL molting of the dsRNA-TsGDH group was decreased by 41.32% (*F = *24.62, *p < *0.001) (Fig. 17).

## Discussion

Metabolism is a chemical reaction process necessary for organisms to maintain life activities. It provides the energy and substances required by parasites and is involved in all life cycle processes, such as parasite invasion of the host, worm molting, and development [25]. Parasites synthesize the substances needed for growth [32]. In the process of development and survival, parasites convert nutrients in the host’s body into the substances they need, thereby achieving growth and reproduction [36]. Metabolism also helps parasites to adapt to environmental changes, maintain a stable physiological state internally, and ensure normal growth and development [18]. Various enzymes play an important role in parasite invasion, molting, and development. Parasite invasion requires a large amount of energy and specific enzymes to break through the host’s natural barriers, such as cell membranes and tight junctions among epithelial cells [5, 47, 49]. Some enzymes such as proteases and lipases help parasites to disintegrate host tissues and promote the invasion process [16, 20]. Additionally, some proteolytic enzymes can also participate in the molting process of nematodes. During the larval molting process, the nematodes need to synthesize new cuticle substances using synthetases and decompose the old outer cuticle through the hydrolysis action of various hydrolytic enzymes to complete the molting process [22, 41]. Therefore, studies on parasite metabolic enzymes are of great significance for gaining a deeper understanding of the biological characteristics, pathogenic mechanisms, and interactions between parasites and their hosts.

GDH, as a catalytic enzyme, is widely present in organisms and utilizes NAD (H) or NADP (H) as its coenzyme for catalytic reactions. Its main function is to participate in the reversible conversion between L-glutamic acid and α-ketoglutaric acid [58]. In the process of amino acid metabolism, the synergistic effect of GDH and aminotransferase plays a crucial role. Firstly, under the action of transaminases, amino acids are transferred to α-ketoglutaric acid to form L-glutamic acid. Subsequently, under the catalysis of GDH, the amino groups in L-glutamic acid are removed to generate ammonia (NH_3_), completing the deamination process in amino acid decomposition metabolism. And the generated α-ketoglutaric acid after deamination enters the tricarboxylic acid cycle, and is thoroughly oxidized and decomposed, and releases energy [10]. These functions make glutamate dehydrogenase a key participant in various biological processes such as intracellular nitrogen metabolism, energy production, and signal transduction [50]. It is of great significance to understand the function of GDH for metabolic pathways, growth, and pathogenesis of parasites, as well as the development of new vaccines and drugs [26, 46].

In this study, TsGDH was cloned and expressed through a prokaryotic expression system and its biological properties and functions were identified. The bioinformatics analysis showed that TsGDH contained an ELFV dehydrogenase domain, with one enzyme active site (Lys 167) and one catalytic site (Asp 209), as well as five substrate binding sites (Lys 131, Lys 155, Thr 256, Asn 295, Ser 424). The presence of these functional sites suggests that TsGDH may play an important role in amino acid metabolism, participating in the redox process of some amino acids. Multi-sequence alignment and evolutionary tree analysis revealed that TsGDH was relatively conserved among different species or genotypes of the genus *Trichinella* and exhibited a more intimate evolutionary relationship among the nine encapsulated *Trichinella* species/genotypes. Furthermore, comparison of TsGDH with the GDH gene sequences of humans and mice revealed that the homology between TsGDH and GDH of humans and mice is only 55.89% and 54.82%, respectively. The results indicated that the evolutionary relationship between TsGDH and the GDH of humans and mice is distant, suggesting that GDH could be used as a drug target against *T. spiralis* without damaging host GDH. Similarly, a homology comparison of the TsGDH gene was also performed with the GDH enzyme of an intestinal tapeworm *Taenia solium*, and an intestinal nematode *Heligmosomoides polygyrus bakeri*. The results showed that the TsGDH amino acid sequence had an identity of 51.62% and 59.59% with the GDH from *T. solium* and *H. polygyrus bakeri*, respectively. *Taenia solium* cysticercus GDH enzymes regulate immune and inflammatory reactions by driving production of prostaglandins PGE2 and IL-10 in monocytes to potentiate Treg induction [38]. The GDH from *H. polygyrus bakeri* suppresses type-2 inflammation via eliciting an anti-inflammatory eicosanoid switch [6]. Our results suggest that TsGDH might have a similar role for regulating immune response and inflammatory reactions during *T. spiralis* infection. Moreover, immunization of mice with rTsGDH induced a specific anti-rTsGDH antibody response, the serum titer of specific anti-rTsGDH IgG reached 1:10^5^, indicating that rTsGDH had good immunogenicity, and also suggesting that rTsGDH was a potential target for anti-*Trichinella* vaccine [45].

The results of qPCR and Western blotting showed that TsGDH was transcribed and expressed in different *T. spiralis* stages, with the higher expression level in the AW stage, suggesting that TsGDH played an essential role during the life cycle of the nematode [30, 39]. TsGDH might be involved in the metabolic activity of *T. spiralis* AWs, providing necessary energy for their survival and reproduction. The IIFT results showed that TsGDH was mainly distributed in the cuticle, stichosome, and embryos of female worms, suggesting that TsGDH might participate in larval molting and female reproduction of the parasite [66, 77, 79].

RNA interference (RNAi), as a gene silencing technique, has a wide range of applications in research on parasites, including parasite function, vaccine development, pathogen control, and drug resistance [53, 69]. RNAi technology can achieve specific gene silencing, allowing researchers to study the function of parasite-specific genes in a targeted manner [4]. RNAi technology is efficient, fast, and easy to operate. Compared to traditional gene knockout or mutation techniques, RNAi technology does not require genome modification and can achieve gene silencing in a shorter period of time, accelerating the research process [56]. To investigate the role of TsGDH in the metabolism, molting, and development of *T. spiralis*, three pairs of specific dsRNAs were prepared to silence TsGDH expression. dsRNA-TsGDH was introduced into the muscle larvae through electroporation [2]. The results showed that when ML were transfected with 50 ng/μL dsRNA-TsGDH for 2 days, the silencing effect on the TsGDH gene was optimal. The natural TsGDH enzyme activity, all the content of ATP, carbohydrates, lipid, and ammonia nitrogen excretion in the treated ML were significantly reduced. The findings indicate that RNAi evidently inhibited the metabolism of sugar, lipids and amino acids. The results of the *in vitro* larva invasion and molting tests showed that after silencing the TsGDH gene, larva invasion and molting were significantly inhibited, indicating that TsGDH played an indispensable role in molting, growth, and development of *T. spiralis* [21, 27].

The results of animal challenge experiments showed that after the mice were orally challenged with the ML treated with dsRNA-TsGDH, the number of collected IIL, 3 and 6 d AWs, NBL production and ML had a significant reduction, further confirming that TsGDH plays an important role in larval invasion of gut mucosa, development, and reproduction of this nematode. In addition, the worm length, natural TsGDH enzyme activity, content of ATP, carbohydrates, lipids, and ammonia nitrogen excretion from the recovered worms showed a significant decrease, indicating that RNAi distinctly reduced the metabolism of various *T. spiralis* stage worms. Furthermore, IIL molting was obviously decreased. Molting is a significant characteristic of the larval growth and development of the nematode [43, 70]. After the TsGDH gene was silenced, *in vitro* and *in vivo* experiments showed that larval molting was inhibited, indicating that TsGDH was an indispensable metabolic enzyme for the metabolism, molting, and development of *T. spiralis,* and it might be a potential candidate target for development of vaccines and drugs [57, 63].

In conclusion, TsGDH was cloned and expressed in this study. TsGDH was expressed in all *T. spiralis* stages with higher expression levels in the AW stage, localized mainly in the cuticle, muscular layer, stichosome, and female intrauterine embryos of this nematode. After silencing TsGDH, larval natural TsGDH enzyme activity was obviously reduced, and its metabolism, molting, growth, and reproduction were also significantly inhibited. The results indicated that TsGDH is an indispensable key enzyme in the life cycle of *T. spiralis*, and participates in metabolism, molting, development, and reproduction. TsGDH might be a potential candidate target for development of anti-*Trichinella* vaccines and drugs.
